# Artificial intelligence and the scientific writing of non-native English speakers

**DOI:** 10.1590/1806-9282.20231291

**Published:** 2024-01-22

**Authors:** Hinpetch Daungsupawong, Viroj Wiwanitkit

**Affiliations:** 1 Private Academic Consultant – Phônhông, Laos.; 2 Chandigarh University – Punjab, India.

Dear Editor,

We would like to share some ideas on the article entitled “The use of artificial intelligence to improve the scientific writing of non-native English speakers^1^.” The research focuses on Chat AI GPT, which is an advanced language model built on the GPT foundation. GPT gained popularity because of its capacity to generate human-like written language. ChatGPT was created for single-turn jobs, but it goes a step further by allowing multi-turn dialogues. This paper investigates the proficiencies and prospective applications of Chat AI GPT in text generation for a variety of purposes. The study used a non-systematic review, which indicates that the literature was not chosen in a rigorous and uniform manner. This could add bias and impair the findings’ reliability and generalizability. The literature review was conducted using only three search phrases (“Artificial Intelligence,” “Scientific Writing,” and “Non-English Speaking”). Because of the restrictive scope, significant research or views that could have provided a more comprehensive understanding of the topic were overlooked. The study does not mention about evaluating the quality or dependability of the included literature. It is difficult to judge the strength of the data offered without analyzing the methodological rigor and validity of the selected studies. While the paper acknowledges the potential benefits of artificial intelligence in scientific writing for non-native English speakers, it neither elaborates about the specific issues that this group faces nor provides a detailed examination of how AI can address those challenges. Finally, it has to concern on the reliability and ethical use of the AI. Without manual input, it is not acceptable. Human user has to verify the content and control the use of AI in the ethical way^2^.

## References

[1] Giglio AD, Costa MUPD (2023). The use of artificial intelligence to improve the scientific writing of non-native English speakers. Rev Assoc Med Bras (1992)..

[2] Kleebayoon A, Wiwanitkit V (2023). ChatGPT, critical thing and ethical practice. Clin Chem Lab Med..

